# Unraveling endothelin-1 induced hypercontractility of human pulmonary artery smooth muscle cells from patients with pulmonary arterial hypertension

**DOI:** 10.1371/journal.pone.0195780

**Published:** 2018-04-12

**Authors:** Jamie L. Wilson, Rod Warburton, Linda Taylor, Deniz Toksoz, Nicholas Hill, Peter Polgar

**Affiliations:** Division of Pulmonary, Critical Care and Sleep Medicine, Department of Medicine, Tufts Medical Center, Boston, Massachusetts, United States of America; Stanford University, UNITED STATES

## Abstract

Contraction of human pulmonary artery smooth muscle cells (HPASMC) isolated from pulmonary arterial hypertensive (PAH) and normal (non-PAH) subject lungs was determined and measured with real-time electrical impedance. Treatment of HPASMC with vasoactive peptides, endothelin-1 (ET-1) and bradykinin (BK) but not angiotensin II, induced a temporal decrease in the electrical impedance profile mirroring constrictive morphological change of the cells which typically was more robust in PAH as opposed to non-PAH cells. Inhibition with LIMKi3 and a cofilin targeted motif mimicking cell permeable peptide (MMCPP) had no effect on ET-1 induced HPASMC contraction indicating a negligible role for these actin regulatory proteins. On the other hand, a MMCPP blocking the activity of caldesmon reduced ET-1 promoted contraction pointing to a regulatory role of this protein and its activation pathway in HPASMC contraction. Inhibition of this MEK/ERK/p90RSK pathway, which is an upstream regulator of caldesmon phosphorylation, reduced ET-1 induced cell contraction. While the regulation of ET-1 induced cell contraction was found to be similar in PAH and non-PAH cells, a key difference was the response to pharmacological inhibitors and to siRNA knockdown of Rho kinases (ROCK1/ROCK2). The PAH cells required much higher concentrations of inhibitors to abrogate ET-1 induced contractions and their contraction was not affected by siRNA against either ROCK1 or ROCK2. Lastly, blocking of L-type and T-type Ca^2+^ channels had no effect on ET-1 or BK induced contraction. However, inhibiting the activity of the sarcoplasmic reticulum Ca^2+^ ATPase blunted ET-1 and BK induced HPASMC contraction in both PAH and non-PAH derived HPASMC. In summary, our findings here together with previous communications illustrate similarities and differences in the regulation PAH and non-PAH smooth muscle cell contraction relating to calcium translocation, RhoA/ROCK signaling and the activity of caldesmon. These findings may provide useful tools in achieving the regulation of the vascular hypercontractility taking place in PAH.

## Introduction

Pulmonary arterial hypertension (PAH) is a devastating condition of the pulmonary vasculature with a high mortality and limited effective treatment [1]. In PAH, pulmonary vascular remodeling and dysregulation of vascular constriction contribute to increased vascular resistance and elevated PA pressure. Human PA smooth muscle cells (HPASMC) are the principal effectors of vascular constriction whose function is dysregulated[2]. The mechanisms contributing to the dysregulated HPASMC contraction are not well understood.

Vasoconstrictors such as endothelin-1 (ET-1) induce contraction in vascular smooth muscle [3]. ET-1 promotes contraction via initial calcium (Ca^2+^) entry into the cytoplasm. ET-1 activates phospholipase C (PLC) which then generates inositol trisphosphate (IP3), leading to intracellular Ca^2+^ release. However, ET-1 has also been reported to increase intracellular Ca^2+^ by stimulating influx through plasma membrane Ca^2+^ channels [4, 5]. In fact, smooth muscle cell contraction has been reported to take place via the extracellular Ca^2+^ route [4]. Once Ca^2+^ enters the cytoplasm, it binds to calmodulin, and the resulting Ca^2+^/calmodulin complex binds to myosin light chain kinase (MLCK). The activated kinase then phosphorylates myosin light chain (MLC) and promotes myosin/actin binding which leads to contraction. ET-1 also phosphorylates MLC phosphatase (MLCP) [6]. This phosphorylation inactivates the phosphatase rendering it unable to dephosphorylate MLC. The MLCP phosphorylation takes place through the action of RhoA/ROCK [7]. Thus, vascular contraction and its maintenance is controlled by a variety of interacting signaling components which fine tune MLC phosphorylation.

Additional elements function to regulate vascular smooth muscle contraction. For example, cofilin in its unphosphorylated state blocks smooth muscle contraction [8]. It has also been reported to bind and disrupt actin leading to inhibition of its polymerization. Caldesmon, a calmodulin binding protein which is functionally analogous to the troponin complex in striated muscle, blocks the myosin/actin interaction leading to impaired cross bridge cycling. The C-terminal end of caldesmon inhibits myosin ATPase activity [9–11]. The actin-binding domain of caldesmon inhibits actinomyosin ATPase [12, 13] by decreasing the rate of inorganic phosphate (Pi) release [14].

This study was designed to obtain a better understanding of the smooth muscle signaling networks participating in the regulation of the contractile responses to vasoactive agents in the vasculature of PAH patients. We chose ET-1 as the vasoactive agent because it is a powerful vasoconstrictor of the pulmonary artery and its concentration is known to be increased within the circulation of PAH patients [15]. We use a novel in vitro procedure to readily and accurately measure smooth muscle cell contraction and assess the input of the signaling elements which regulate it. This real-time impedance technique has recently been validated for measurement of cellular contraction in human primary bronchial smooth muscle cells by Bravo and coworkers [16]. Our results illustrate that HPASMC obtained from subjects with PAH maintain contraction mechanisms of the non-PAH HPASMC but exhibit alterations in calcium influx [17], sensitivity to ROCK1/ROCK2 and an increase in the expression of proteins making up the contractile constituents [18]. Findings on the source of cytoplasmic [Ca^2+^], the participation of caldesmon in the regulation of contraction, alterations in RhoA/ROCK sensitivity and the non-involvement of cofilin and LIM kinase (LIMK) in PAH smooth muscle contraction open new avenues to modulation of the excessive contraction taking place in pulmonary arterial hypertension.

## Materials and methods

### Chemicals and reagents

Endothelin-1 (ET-1) and sarafotoxin S6c were purchased from American Peptide (Sunnyvale, CA). Pharmacological inhibitors nitrendipine, nifedipine, thapsigargin, U0126, mibefradil and Y-27632 were purchased from Cayman Chemicals (Ann Arbor, MI). Pharmacological inhibitor BI-D1870 was purchased from Enzo Biochem (Framingdale, NY). For siRNA knockdown experiments, siRNAs were purchased from Santa Cruz Biotechnology (Santa Cruz, CA) and Lipofectamine 2000 and Opti MEM from Life Technologies (Carlsbad, CA). MMCCP were synthesized by 21st Century Biochemicals, Inc (Marlboro, MA).

### Antibodies

Primary antibodies against p-cofilin 1 (Ser-3) (#CP1151) and cofilin 1 (N-terminus) (#CP1131) were purchased from EMC Biosciences (Versailles, KY). Additional, primary antibodies against ROCK1 (#sc-374388) or ROCK2 (sc-5561) were purchased from Santa Cruz Biotechnology (Dallas, TX). Beta actin (#3700) and secondary rabbit and mouse antibodies (#7074, #7076) were purchased from Cell Signaling Technologies (Danvers, MA).

### Cell culture

Human pulmonary artery smooth muscle cells (HPASMC) were a generous gift from the Cleveland Clinic (Cleveland, OH) and were derived and maintained as described by Comhair et al. 2012 [19]. Details about the handling and culture of the cells and their donors can be found in a previous communication, Yu et al. 2013 [17]. Briefly, cells were isolated from elastic pulmonary arteries (>500-μm diameter) from explanted lungs of PAH patients and non-PAH donors. Two additional primary HPASMC were purchased from Lonza (Wakersville, MD) (Cat# CC2581, Lot# 369143) and Cell Applications Inc. (San Diego, CA) (Cat# 352-05a, Lot# 1487). Primary cultures of passages 6–10 were used in experiments and smooth muscle phenotypes were confirmed via immunostaining for alpha-smooth muscle actin. Specific information about the cell donors used in this study is given in **S1 Table**.

### HPASMC constriction measurements

Smooth muscle cell contraction determination in culture has previously been performed by time lapse photography, cellular force production and collagen gel assay [20–22]. Each of these procedures has limitations such as being subject to bias due to irregular cell morphology, being technically challenging or semi-quantitative. Electrical impedance measurements using an xCelligence RTCA DP instrument (Acea Biosciences, San Diego, CA) has enabled us to measure HPASMC contraction rapidly, noninvasively and quantitatively [17, 23, 24]. Measurements of real-time cell contraction were performed in a 5% CO_2_ incubator at 37°C [25]. HPASMC at 80–90% confluence were seeded at a density of 4500 cells/well on an E-plate 16 (ACEA Biosciences) in growth medium at 37°C [16]. All controls and treatments in these experiments were performed in triplicate. After seeding, the cells were allowed to attach for 24 h. Following attachment, the medium was changed to that containing 0.2% fetal bovine serum and the cells were pre-incubated for another 24 h. Each well was pretreated for 1 h with pharmacological inhibitor, MMCPP, or vehicle before addition without (CON) or with agonist. To record HPASMC constriction, the instrument measured cell impedance every 30 seconds for 1.5 h. For experiments involving siRNA knockdown of ROCK, wells were transfected with 3 pmol of negative control siRNA (sc-37007), human ROCK1 siRNA (sc-29473) or human ROCK2 siRNA (sc-29474) in medium containing Lipofectamine 2000 and Opti MEM according to manufacturer’s instructions.

The measured change in impedance reflects a temporal cytoskeletal rearrangement and an overall change in morphology. Based on the magnitude and duration, these agonist induced decreases in impedance reflect a cumulative, temporary, contracted morphology of the HPASMC akin to *in vivo* HPASMC contraction during blood vessel constriction. These impedance measurements are expressed as the cell index. Normalized cell index is the cell index value at a time point divided by the cell index at the starting point.

### Western blots

Cells were seeded in 6-well cell culture plates until reaching confluence. At that point, the medium was replaced with serum-free medium and the cultures were incubated overnight at 37°C at 5%CO_2_. The cells were then pretreated with or without inhibitors for 1 h before treatment with ET-1. The cells were then lysed using 100 μl RIPA buffer (150 mM NaCl, 1.0% Igepal CA-630, 0.5% sodium deoxycholate, 0.1% SDS, 50 mM Tris, pH 8.0 (Sigma, St Louis, MO) containing both complete protease inhibitor cocktail and PhosSTOP phosphatase inhibitor cocktail (Roche Applied Science, Indianapolis, IN). Protein was isolated from each well. Total protein was quantified using BCA assay. Equal amounts of protein were loaded and electrophoresed on SDS-PAGE. Proteins were then transferred onto Immobilon-P 0.45 um PVDF membrane (EMD Millipore, Darmstadt, Germany) at 100 V, 4°C for 1 hour. The PVDF membranes were blocked at room temperature for 1 hour with 5% powdered milk in TBS-T (20 mM Tris, 150 mM NaCl, 0.1% Tween 20, pH 7.6) before incubating with primary antibody overnight at 4°C diluted in TBS-T with 5% BSA. Blots were washed again in TBS-T followed by 1 h incubation with the corresponding anti-rabbit or anti-mouse 2° Ab (Cell Signaling, Danvers, MA) diluted 1:2000 TBS-T. Blots were then washed in TTBS-T and developed using SuperSignal West Pico PLUS Chemiluminescent Substrate (Thermo Scientific, Rockford, IL) for 1 min before imaging on a Fluor Chem E system (Protein Simple, San Jose, CA).

### Statistics

Results, as applicable, are presented as n = number of biological samples, mean with standard deviations and were evaluated for statistical significance using a student’s t-test or as stated in the legend.

## Results

### Effect of vasoactive peptides on HPASMC contraction

**Fig 1:**
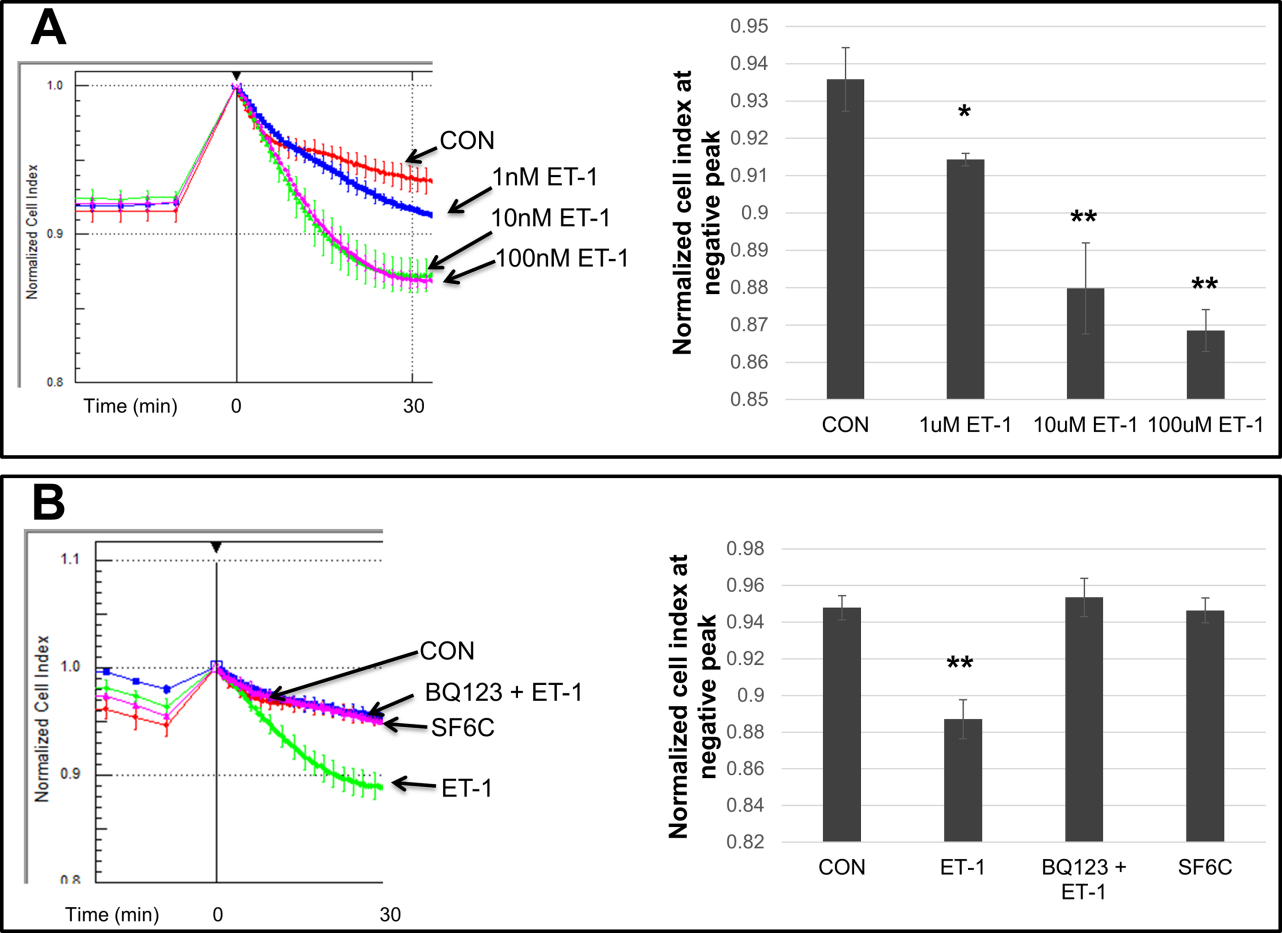
Detection of ET-1 induced contraction in HPASMC by electrical impedance. Electrical impedance was measured as described in the methods section. Triplicate wells were used with or without (CON) 1 nM, 10 nM or 100 nM ET-1 (A). The line graphs represent the average normalized cell index across triplicate wells and the error bars are the standard deviation at each time point. The bar graphs represent the average negative peak for each triplicate and the error bars are the standard deviation at that time point. Triplicate wells treated with vehicle (CON), 1 uM BQ123 plus 10 nM ET-1, 10 nM ET-1 or 10 nM of sarafotoxin (SF6C) were measured as well (B). This figure illustrates a non-PAH HPASMC (Control-1) strain that is representative of results obtained from various HPASMC strains. * p< 0.01 vs CON; ** p< 0.001 vs CON.

**Fig 2:**
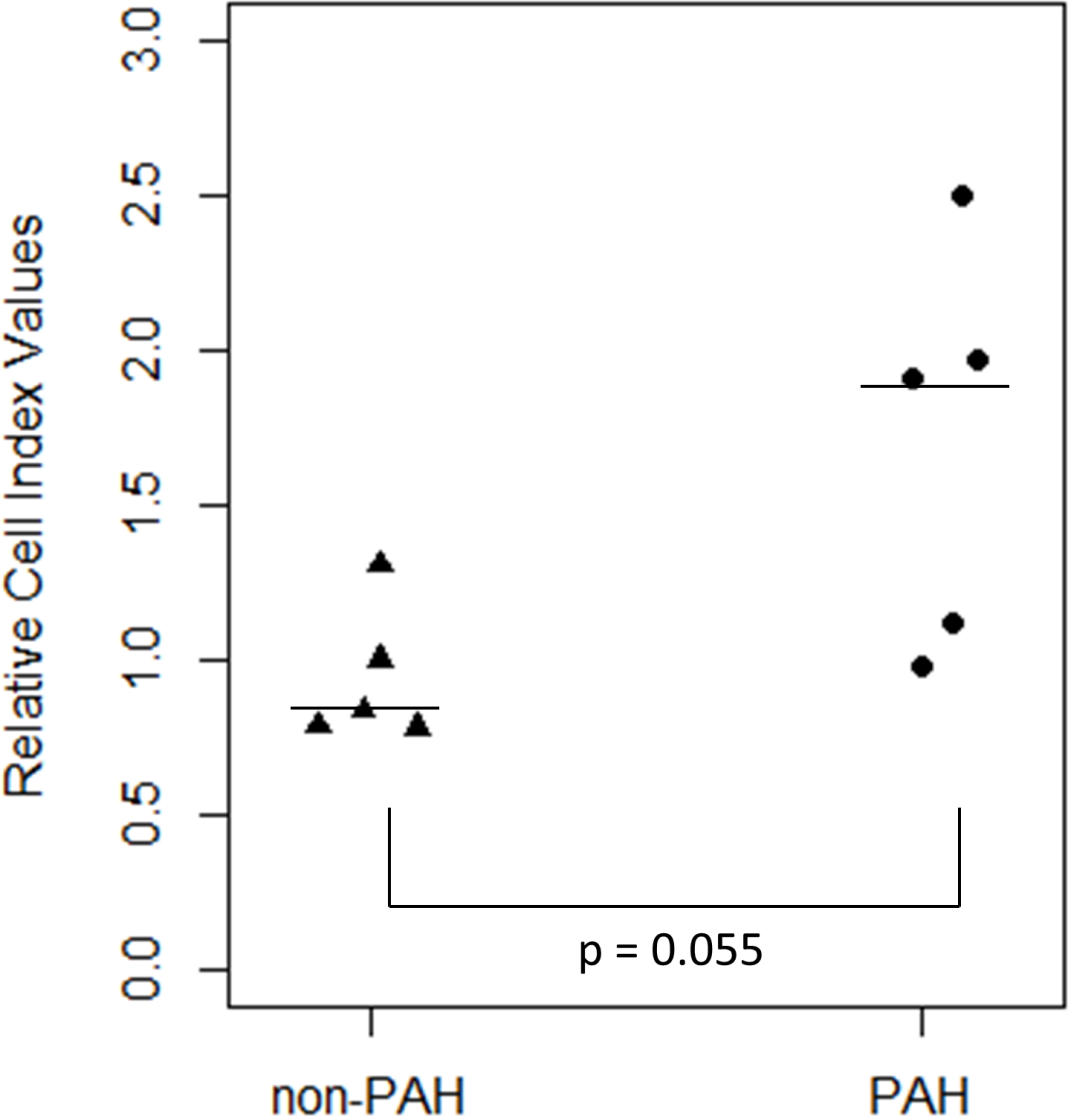
Dot plot comparing ET-1 induced contraction magnitudes in non-PAH and PAH HPASMC. Contraction magnitudes were calculated for non-PAH (n = 5) and PAH (n = 5) HPASMC by taking the maximum negative peak value of each sample and dividing by one of the non-PAH samples giving a relative cell index value. Vertical dots indicate relative cell index for each non-PAH (triangle) and PAH (circle) HPASMC sample. The horizontal line represents the median of the group. A Welch’s two sample t-test assuming unequal variance was performed between the non-PAH and PAH groups; p-value = 0.055.

Endothelin-1 (ET-1) is a powerful vascular smooth muscle contractor [4, 26]. Its expression increases in patients with pulmonary hypertension [27]. Consequently, ET-1 receptor blockers have been used to treat PAH [28]. HPASMC contraction was confirmed in response to this vasoactive effector in a dose-dependent manner, **Fig 1A**. As shown, the contraction began immediately after ET-1 addition and peaked in approximately 30 minutes in both the PAH and non-PAH HPASMC. Since the impedance measurement following ET-1 addition takes place in minutes, any effect due to cell proliferation can be excluded as changes in cell number which could alter the impedance measurements would take hours if not days in HPASMC. Maximum dosage for the contraction was reached at 10 nM ET-1 in both PAH and non-PAH HPASMC. ET-1 binds to two receptors, ETA and ETB. Both receptors have been reported to elicit smooth muscle contraction [29–31]. However, the HPASMC contractile response to ET-1 was completely abolished by the ETA receptor antagonist BQ123 as shown in **Fig 1B**. Additionally, as shown in the same figure, the ETB specific agonist, sarafotoxin S6C, did not induce contraction. Time lapse microscopy was performed as a confirmation of the ET-1 induced HPASMC contraction. **S1 Fig** shows this contraction from time 0 until 40 minutes after ET-1 addition. As illustrated, the HPASMC pulled in their cytoplasm in response to 100 nM ET-1. When comparing magnitudes of ET-1 induced contractions between PAH and non-PAH HPASMC, we typically found HPASMC from PAH individuals had 1.5 to 2 two-fold higher impedance changes compared to non-PAH (**Fig 2**). A direct comparison of a representative non-PAH and two PAH (hereditary and idiopathic) ET-1 induced contraction impedance profiles is shown in **S2 Fig**. However, although the median relative ET-1 induced contraction magnitudes were higher in the PAH group compared to non-PAH group, their differences with respect to the mean were not statistically different (p-value = 0.055) (**Fig 2**).

**Fig 3:**
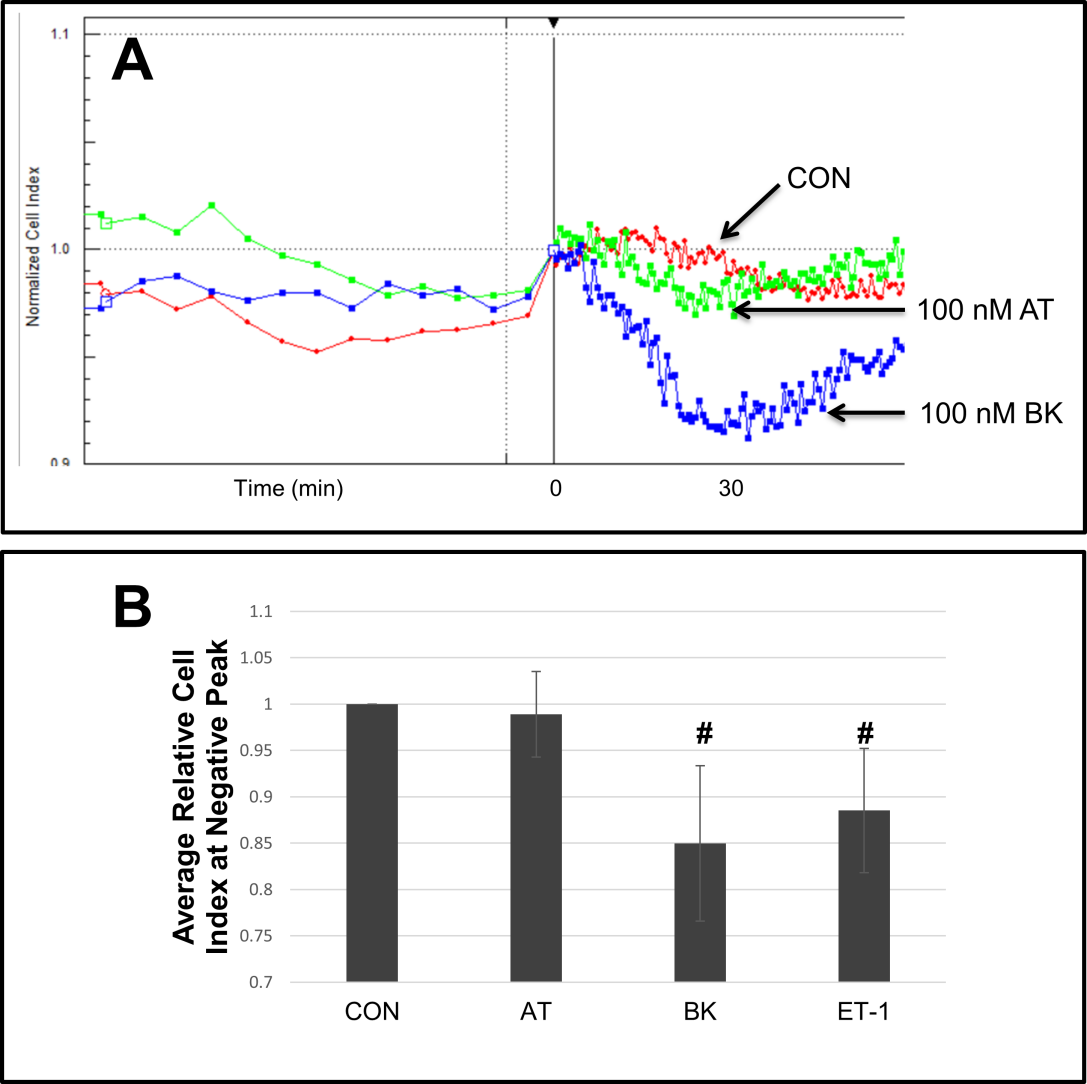
Effect of vascular effectors, bradykinin and angiotensin, on HPASMC contraction. A representative HPASMC sample (Control-5) showing the average normalized cell index of triplicate wells treated without (CON) or with 100 nM bradykinin (BK) or 100 nM angiotensin II (AT) (A). Bar graph where bar heights represent average relative cell index at negative peak for n = 4 HPASMC strains. The error bars represent standard deviation. Relative cell index at negative peak was calculated by dividing the minimum cell index of the treatment group (AT, BK or ET-1) by the control group (CON) at that same time point. This was done for each HPASMC cell strain. Then, an average and standard deviation were calculated across n = 4 biological replicates. * p< 0.01 vs CON.

Bradykinin is generally a smooth muscle dilator in intact tissue. This dilatory effect takes place via the release of nitric oxide (NO) by endothelial cells in response to bradykinin [32]. However, PAH is often accompanied by endothelial dysfunction [33]. Here we explored this circumstance with bradykinin contractile function in the absence of endothelial cells. As illustrated, in the absence of the endothelium BK, at 100 nM, induced contraction similar to that of ET-1 (**Fig 3**). Interestingly, angiotensin II (AT) which is often a powerful contractile agent showed only a limited contractile effect in HPASMC as shown at its maximal effect (**Fig 3A**). Across multiple HPASMC derived from different patients (strains) this effect was not significant (**Fig 3B**).

### Lack of participation of LIMK and cofilin in HPASMC contraction

**Fig 4:**
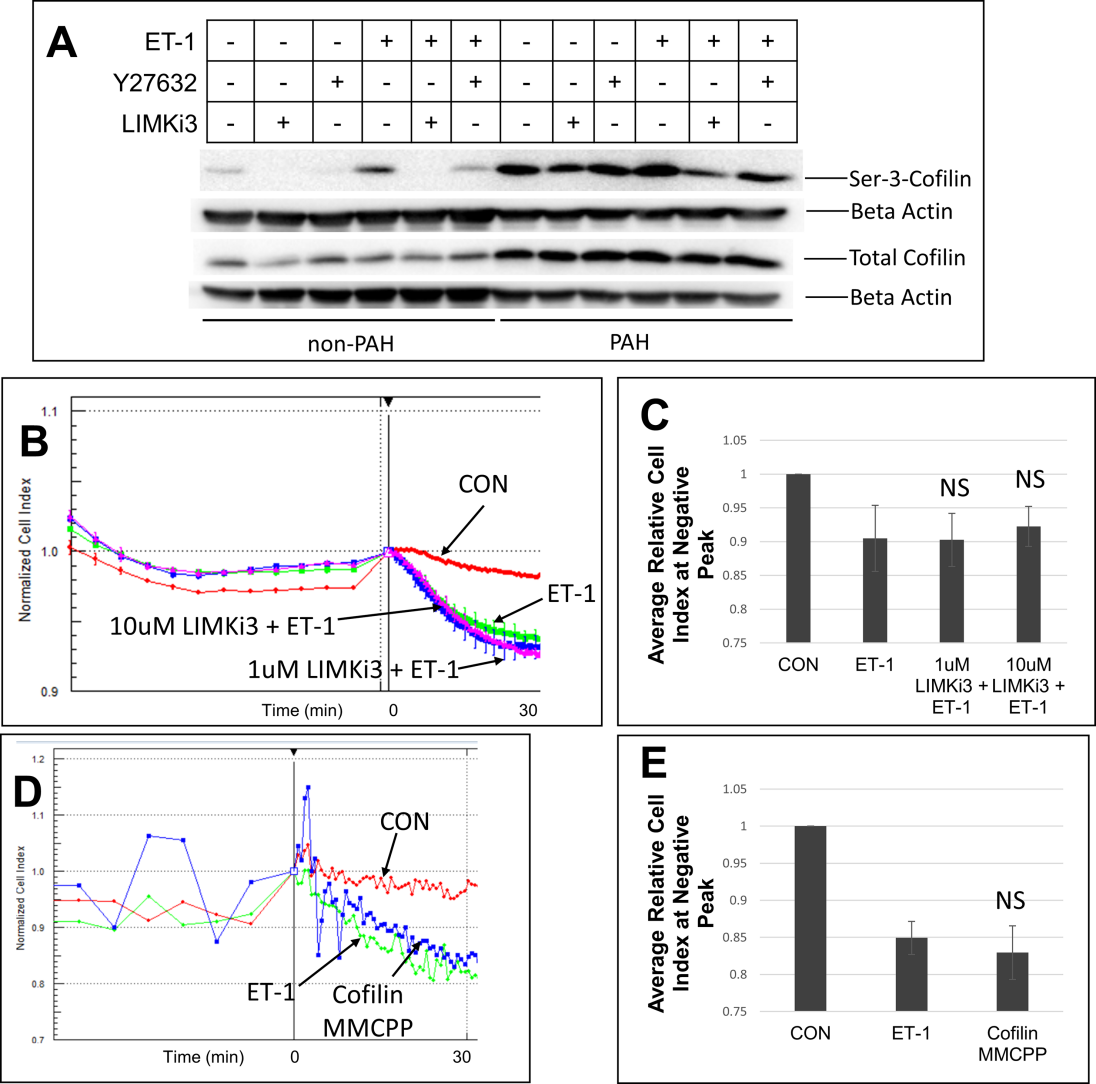
LIMK effect on ET-1 promoted contraction in HPASMC. Representative western blot of non-PAH (Control-1) and PAH (PAH-3) HPASMC proteins probed for p-cofilin, total cofilin and beta actin after pretreatment with or without 10 uM LIMi3 or 10 uM Y27632 and treatment with or without ET-1. Each sample had 22 ug of protein loaded per well on two separate gels which later were probed for p-cofilin and beta actin or total cofilin and beta actin (A). A representative HPASMC sample (Control-1) showing the average normalized cell index of triplicate wells pretreated with or without LIMKi3 1 h before treatment without (CON) or with ET-1 (B, C). Another representative HPASMC (PAH-1) showing the result of pretreated with MMCPP targeted against cofilin (Cofilin MMCPP) for one h before addition of ET-1 (D, E). Bar graphs represent average relative cell index at negative peak for n = 4 HPASMC strains and the error bars are the standard deviation (C, E). NS = not significant vs ET-1.

We investigated participation of actin interactive regulatory elements which are not Ca^2+^ associated in vascular smooth muscle contraction. Two of the elements, LIMK and cofilin, were previously shown by us to participate in HPASMC migration [34]. LIMK’s function has been linked to the phosphorylation of cofilin leading to p-cofilin uncoupling from actin thus allowing actin activity [35]. **Fig 4A** shows ET-1 promoting cofilin phosphorylation at Ser-3 and this action is greatly inhibited by LIMKi3 in both non-PAH and PAH HPASMC. The ROCK inhibitor Y27632 also reduces cofilin phosphorylation but less than LIMKi3. The PAH HPASMC showed higher expression of p-cofilin and total cofilin protein compared to the non-PAH HPASMC. However, **Fig 4B and 4C** illustrate that inhibition with LIMKi3 has no effect on contraction in HPASMC. To further confirm the lack of this cascade in the HPASMC we directly examined the action of cofilin in the process of contraction. Motif mimicking cell permeable peptides (MMCCP) were constructed against cofilin to block its phosphorylation [34, 36, 37]. Similar peptides were previously shown to inhibit LIMK’s ability to phosphorylate cofilin *in vitro* and *in vivo* [37, 38]. Nonsense MMCCP were simultaneously generated. In **Fig 4D and 4E**, we confirm the lack of cofilin participation in HPASMC contraction using these MMCPP. The same MMCPP were shown previously to inhibit migration in PAH and non-PAH HPASMC in response to PDGF [34].

### Participation of ROCK in HPASMC contraction, an altered response in PAH

**Fig 5:**
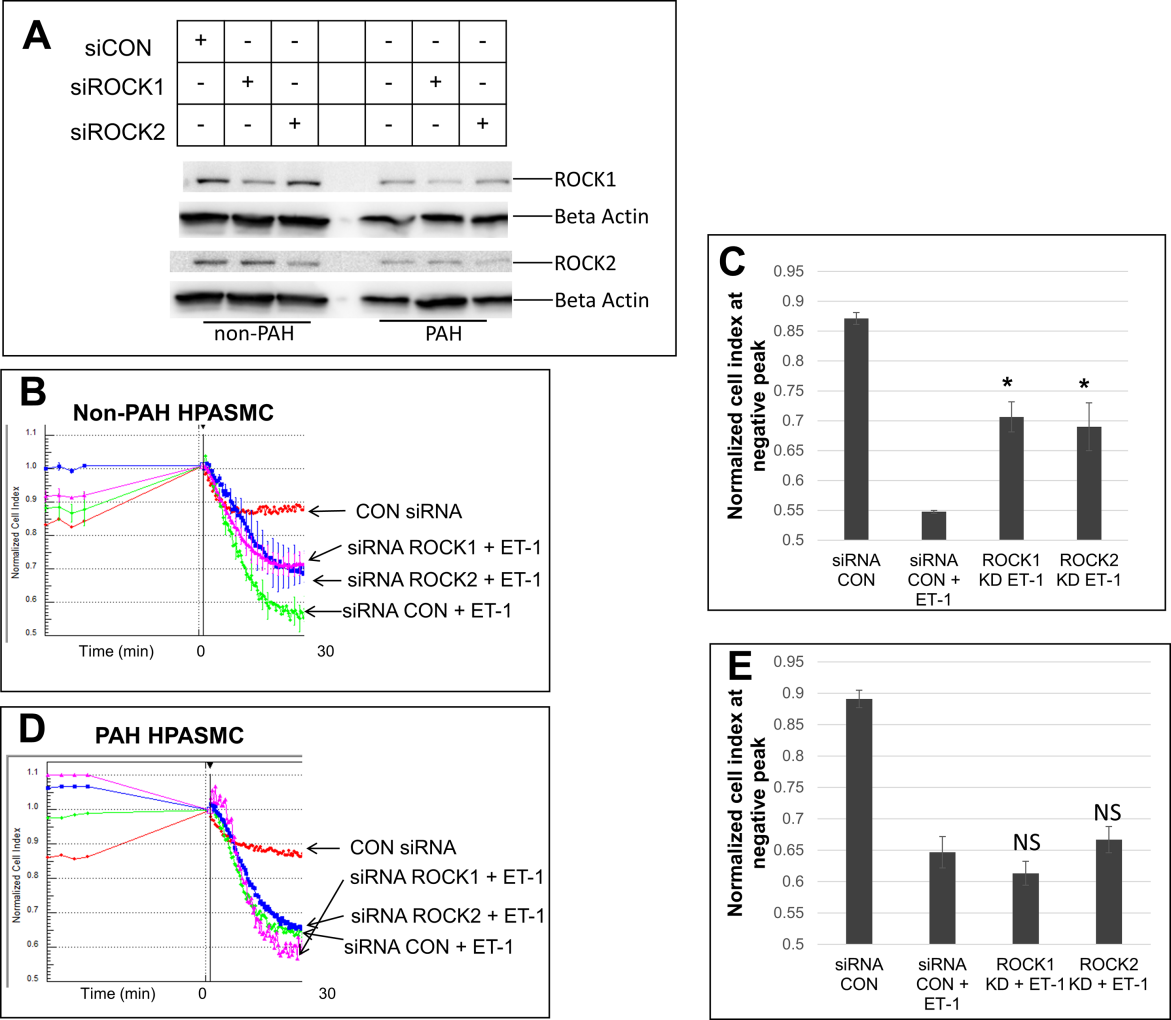
Effect of ROCK siRNA knockdown on the ET-1 induced contraction in non-PAH and PAH HPASMC. Western blots showing protein levels of ROCK1 and ROCK2 after siRNA knockdown with ROCK1, ROCK2 or control siRNA in non-PAH (Control-1) and PAH (PAH-1) HPASMC. Thirty ug of total protein was loaded for non-PAH samples and 15 ug of total protein was loaded for the PAH samples (A). Representative impedance experiments showing the average of triplicate wells which were pretreated with negative control (CON) siRNA, ROCK1 siRNA or ROCK2 siRNA and then treated with or without ET-1. The line graph represents the average normalized cell index across triplicate wells and the error bars are the standard deviation (B, D). These values at the negative peak of the contraction are illustrated with bar graphs (C, E). ROCK1 KD, ROCK1 knockdown with ROCK1 siRNA; ROCK2 KD, ROCK2 knockdown with ROCK2 siRNA; NS = not significant vs siRNA CON + ET-1; *p< 0.01 vs siRNA CON + ET-1.

While the LIMK/cofilin route is not participating in HPASMC contraction three very important signaling proteins known to respond to ET-1 are, RhoA and its downstream kinases ROCK1 and ROCK2. Both ROCK1 and ROCK2 siRNAs knocked down their respective proteins by about 50% in both non-PAH and PAH HPASMC as shown by western blot in **Fig 5A**. Both ROCK siRNAs knockdowns decreased ET-1 constrictive response in non-PAH HPASMC (**Fig 5B and 5C**). However, these knockdowns did not affect ET-1 induced contractions in the PAH HPASMC (**Fig 5D and 5E**).

**Fig 6:**
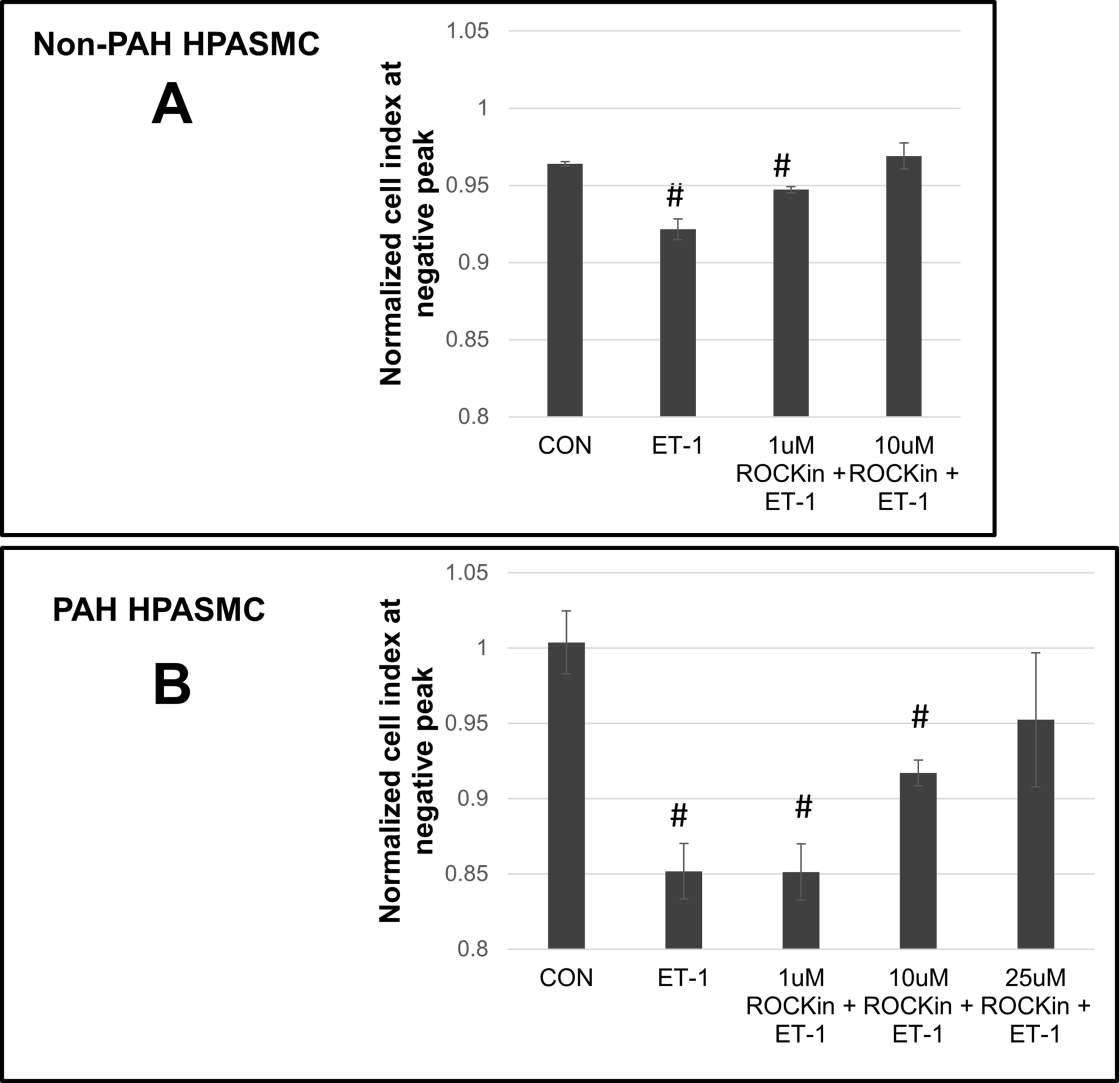
Effect of ROCK inhibitor on the ET-1 induced contraction in non-PAH and PAH HPASMC. Representative experiments showing triplicate wells which were pretreated without or with 1, 10 uM or 25 uM Y27632 (ROCKin) for 1 h and then with or without ET-1 in non-PAH (A) and PAH (B) HPASMC. These values at the negative peak of the cell index are illustrated with bar graphs with error bars representing standard deviations. #p< 0.05 vs CON.

Resistance to inhibition of ROCK activity in the PAH HPASMC is further illustrated using the pan ROCK inhibitor, Y27632. The inhibitor dose-dependently decreased ET-1 induced cell constriction in non-PAH HPASMC at 1 uM with a complete abrogation at 10 uM (**Fig 6A)** while it equivalently affected ET-1 induced cell constriction in PAH HPASMC at a much higher inhibitor concentration, 10 and 25 uM respectively (**Fig 6B**).

### Role of caldesmon in HPASMC contraction

**Fig 7:**
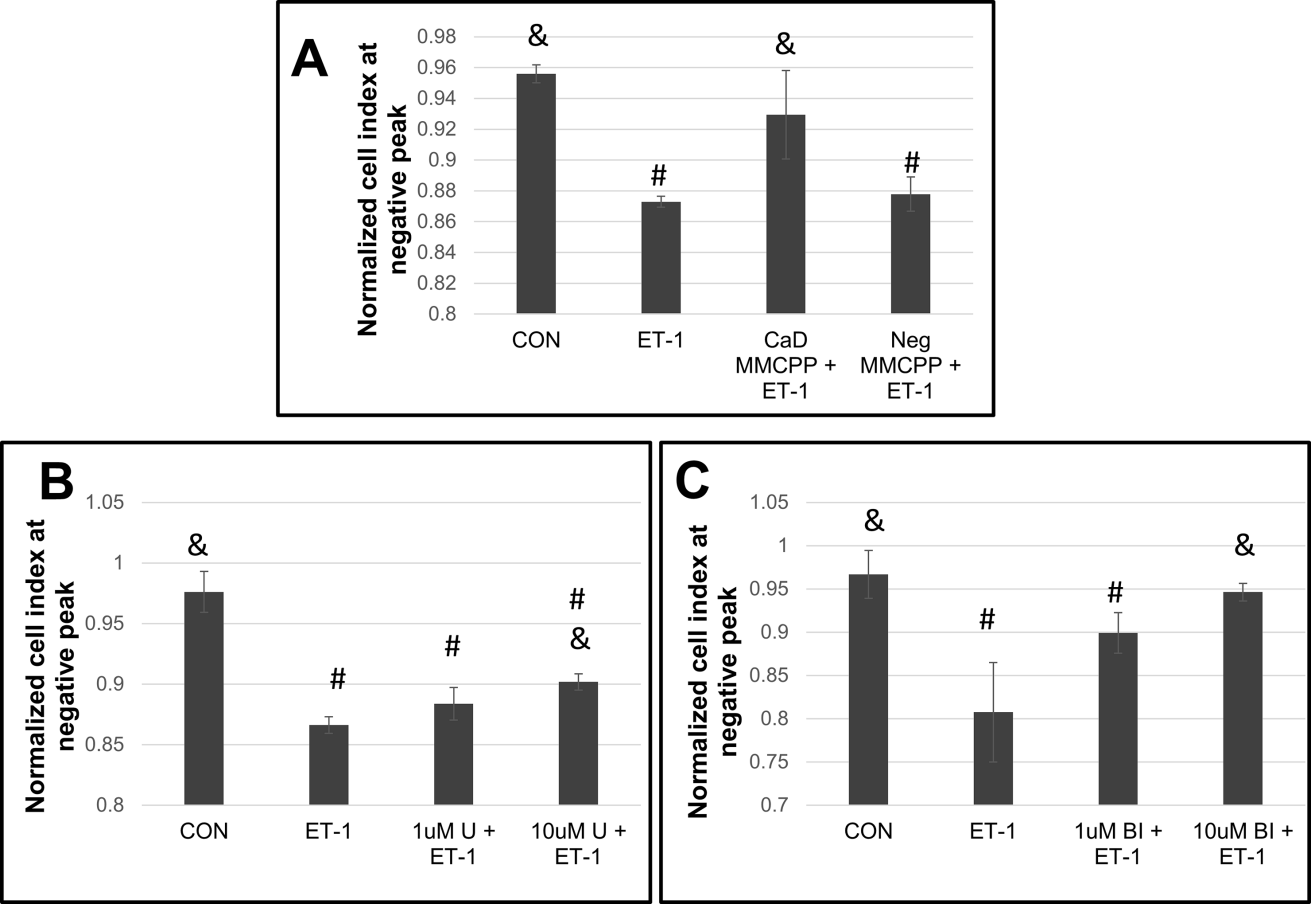
Effect of caldesmon, MEK and p90RSK inhibition on ET-1 induced cell contraction. Representative experiments showing bar graphs with standard deviation error bars for caldesmon activity inhibition with a caldesmon MMCPP (CaD MMCPP) or negative control MMCPP (Neg MMCPP) (A), MEK inhibition with U0126 (U) (B) and p90RSK inhibition with BI-D1870 (BI) (C). All inhibitors were pre-incubated with the cells for 1 h before addition of ET-1. #p< 0.05 vs CON; &p< 0.05 vs ET-1.

Caldesmon has been reported to participate in vascular smooth muscle contraction [39, 40]. Its inhibitory interaction with actin is regulated by MAP kinases (p38, ERK, JNK) [41–43]. Phosphorylation of ERK at Ser789 leads to caldesmon inactivation and consequent myosin/actin interaction [44, 45]. Caldesmon's interaction with actin is inhibitory to contraction in its unphosphorylated form [40]. To block caldesmon's action Huang & Wang, 2006, constructed a MMCPP which was attached to the penetratin carrier peptide [46]. This construct blocked caldesmon promoted actin/myosin interaction in gizzard SMC [46]. The mechanism of the peptide action was not determined but projected to inhibit the phosphorylation of caldesmon at ERK [46]. In experiments herein, we attached this peptide to the SynB3 carrier peptide. As reported by Huang & Wang, 2006, we also observed an inhibition of ET-1 promoted contraction of HPASMC. To confirm the inhibitory function of this MMCPP, nonfunctional control MMCCP were simultaneously generated with Asp replacing Ser [34]. As illustrated in **Fig 7A,** MMCPP targeting caldesmon function [46] effectively blocked ET-1 promoted HPASMC contraction while a control peptide did not. To further test a role of caldesmon and MAP kinases in HPASMC contraction, the action of MAP kinases was blunted with an inhibitor of MEK (U0126), an upstream kinase of ERK (**Fig 7B)**. Inhibiting the action of MEK also partially blunted the contractile response to ET-1. Further evidence of the participation of this cascade on contraction of HPASMC was derived by inhibiting the activity of p90RSK (BI-D1870), a kinase downstream of ERK, which also inhibited ET-1 induced contraction (**Fig 7C).**

### Source of calcium in ET-1 and bradykinin promoted contraction

**Fig 8:**
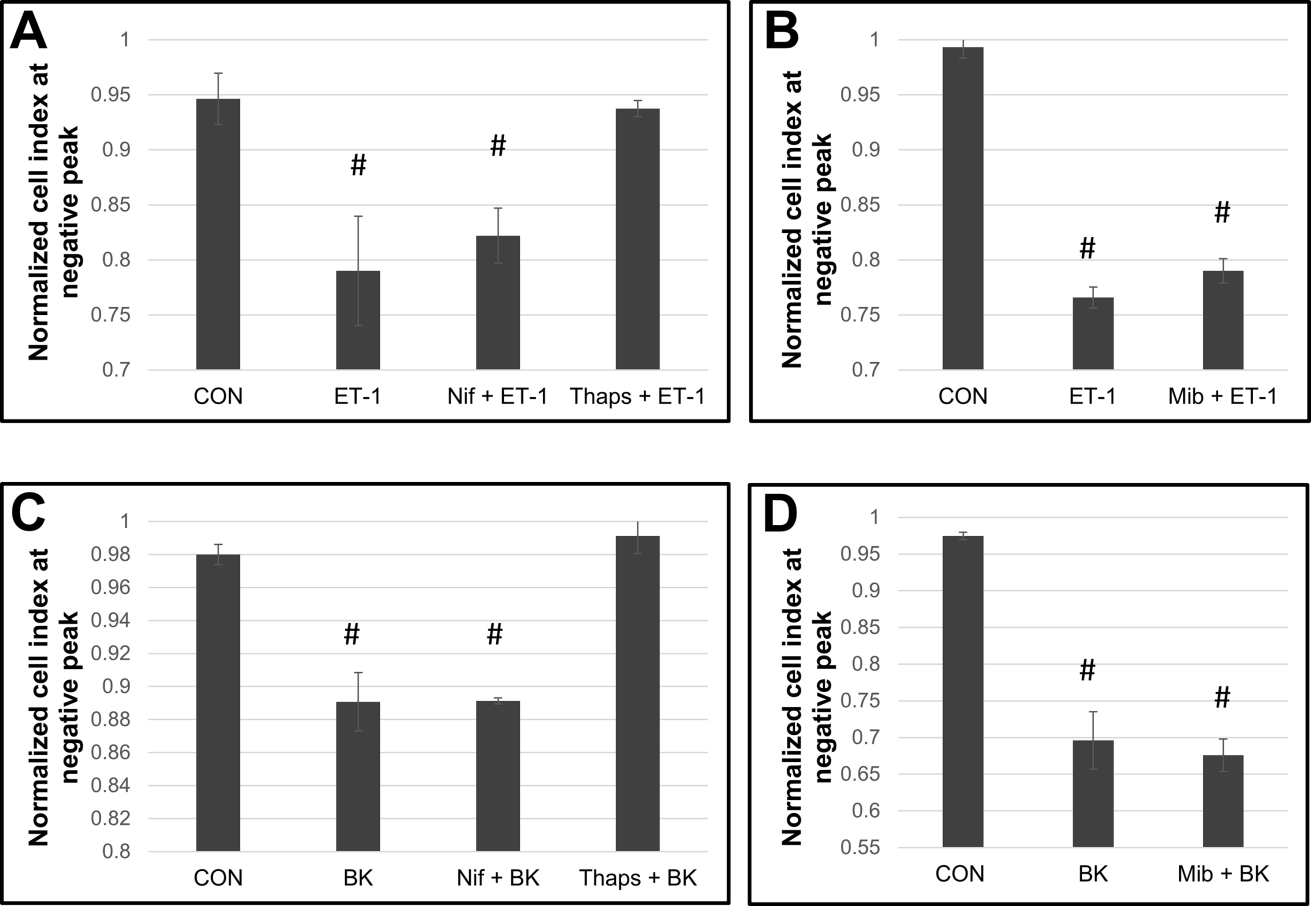
The effect of calcium channel blockers on ET-1 and bradykinin induced cell contraction. Representative experiments showing bar graphs with standard deviation error bars for the effects of 10 uM nifedipine (Nif), 10 uM thapsigargin (thaps) (A, C), or mibefradil (Mib) (B, D) on ET-1 (A, B) or bradykinin (BK) (C, D) induced cell contraction. All inhibitors were pre-incubated with the cells for 1 h before addition of ET-1 or BK. #p< 0.05 vs CON.

Signal promoted vasoactive SMC contraction is achieved through the interaction of Ca^2+^ sensitive and Ca^2+^ independent paths. In a previous communication, we demonstrated that cytoplasmic Ca^2+^ levels are increased approximately two-fold in PAH cells in response to ET-1 compared to non-PAH cells [17]. However, it was not clear as to the source of this Ca^2+^ influx in the two HPASMC groups. Furthermore, reported data have pointed to both extracellular and intracellular Ca^2+^ stores as contributing to the contraction [4]. Here, we examined the contributions of extra and intracellular Ca^2+^ cytoplasmic inflow to ET-1 and bradykinin induced contraction of HPASMC by using specific Ca^2+^ flux blockers. The pharmacological inhibitors nifedipine and mibefradil were used to block L and T plasma membrane Ca^2+^ channels, respectively. Preincubation with thapsigargin permanently depleted the sarcoplasmic reticulum of Ca^2+^ prior to incubation with ET-1. Pretreatment with thapsigargin blocked contraction in response to either bradykinin or ET-1 (**Fig 8A and 8C**). The L (**Fig 8A and 8C**) or T (**Fig 8B and 8D**) type channel blockers had no effect on ET-1 or bradykinin contraction. Both non-PAH and PAH cell strains demonstrated that sarcoplasmic Ca^2+^ efflux was the source of the (Ca^2+^) for both ET-1 and bradykinin.

## Discussion

In this communication, we investigate the expression, action and interaction of signals known to be important in pulmonary vascular smooth muscle contraction. ET-1 was used primarily because it is a powerful vasoconstrictor involved in the pathophysiology of PAH and activates typical G-protein receptors[47]. While the degree of contraction of PAH cells in response to ET-1 was well above that of non-PAH HPASMC, we found that the contractile responses of the PAH and non-PAH PASMC strains to ET-1 signaling were mechanistically very similar (within the limits of the cell sample number available). This finding is very different from the dysregulated migration in PAH previously reported by us and others [2, 34, 48] and the dysregulated proliferation [49–53] of PASMC from PAH patients and hypoxic rodent models.

Here, we demonstrate that both PAH and non-PAH HPASMC contract not only in response to ET-1 but also bradykinin in the absence of the endothelium. The contractile response to bradykinin may be an important observation because endothelial damage is prevalent in PAH; with dysfunctional NO metabolism setting the stage for an alternate, pathologic, vasoconstrictive action of bradykinin [54, 55]. Interestingly while angiotensin did not appear to be involved in HPASMC contraction, it has been reported to be involved in migration of SMC [56] and contraction of rat aortic smooth muscle cells [57].

As discussed in the introduction, the regulation of smooth muscle contraction depends on an interplay among [Ca^2+^]_i_/calmodulin, actin, myosin and associated proteins/kinases/phosphatases which alter the action of actin through diverse phosphorylations. These proteins are vasoactive agent receptor responsive and participate in contractile effector signaling mechanisms. Clearly, the MAP kinases participate in the regulation of contraction as illustrated in **Fig 7**. Inhibiting MEK activity and its downstream targets ERK and then p90RSK effectively modulated ET-1 induced contraction. This MAP kinase sensitive regulation appears to be related to the actions of caldesmon [42]. Possibly through ERK or JNK sensitive phosphorylation at several serine/threonine sites [58]. A peptide sequence reported by Huang & Wang, 2006 [46] covalently linked to a SynB3 carrier peptide was synthesized as a MMCPP to modulate caldesmon activity. This MMCPP limited the dysregulated PAH HPASMC migration [34] as well as the ET-1 promoted contraction herein (**Fig 7A**). Thus, whereas caldesmon modulates both migration and contraction in the HPASMC, our results show that cofilin only influences migration [34]. Other studies on vascular smooth muscle cells have also confirmed caldesmon’s role in contraction and migration [39–41, 59]. Interestingly, Dai et al. reported that both ET-1 and angiotensin II induced constrictive responses in canine PA rings. However, while ET-1 increased phospho-cofilin in canine PA rings and rat PASMC, angiotensin II did not [60]. This implies subtle differences in pulmonary arterial vessel contraction mechanisms that are agonist and cell specific. These authors also reported that cofilin was upregulated in the pulmonary arteries of monocrotaline-treated rats and likely contributing to growth and migration of the PASMC [35].

ROCK is a key contributor to the small GTPase RhoA mediated sensitization of smooth muscle contraction [3]. While ROCK participates in the regulation of contraction of both the non-PAH and PAH cells, clearly the PAH cells demonstrate ROCK responsive constriction which is markedly more resistant to modulation through the inhibition of RhoA/ROCK activity. It took approximately two and half fold greater concentration of ROCK inhibitor Y27632 to achieve blockage of ET-1 stimulated contraction in PAH cells. Additionally, siRNA against either ROCK1 or ROCK2 proved ineffective in blocking ET-1 stimulated PAH HPASMC contraction while effectively blocking contraction in non-PAH cells. Cellular RhoA level measurements are difficult to obtain in primary cells such as HPASMC because of the large cell number needed for the RhoA pulldown assay. However, the observations obtained with regard to the increased sensitivity to RhoA in PAH cells go in hand with our mass spectrometry results showing that the expression of Rho GTPase-activating protein-1 is approximately 1.5 fold higher in PAH HPASMC relative to non-PAH HPASMC (unpublished data). While the overall mechanism(s) of these alterations taking place in PAH is presently not clear a dysregulation of ROCK activity and a defective response to NO in PAH have been described [54, 55]. However, within the spectrum of the experiments herein NO action canceling the activity of RhoA via its synthesis by the endothelium is not available. Additionally, with a comparatively greater than two-fold increase in Ca^2+^ influx in PAH HPASMC following ET-1 stimulation [17] the PAH HPASMC need considerably less RhoA/ROCK to carry out and then sustain the ET-1 stimulated contraction [4]. This critical phenomenon of PAH is likely related to RhoA/ROCK having a functional effect at much lower active level in PAH than in non-PAH HPASMC.

A strategy for ameliorating the effects of PASMC hyperconstriction in PAH patients with ROCK inhibitors such as fasudil has been attempted. However, these studies have shown only marginal effectiveness in lowering pulmonary arterial pressure in PAH patients [61–63]. Our results demonstrate a reason for this phenomenon. Considerably higher ROCK inhibitor concentrations are necessary to reach control of ROCK activity in PAH. An increase in ROCK activity in PAH has been suggested by others [64, 65].

[Ca^2+^]_i_ is the primary factor in contraction and likely a central participant in the more potent contraction of PAH smooth muscle cells. Thus, another objective of this study was to determine the source of [Ca^2+^]_i_ which initiates contraction by vasoactive agents. The source of this flux with regard to ET-1 and other vasoactive peptides has been reported to be both extracellular and intracellular [4]. Voltage regulated transporters have been linked to this process [4]. Our results show that the contraction following stimulation of PAH HPASMC with ET-1 or bradykinin is dependent on active Ca^2+^ efflux from the endoplasmic reticulum (ER) and not from the extracellular space. As illustrated, this efflux from the ER is directly linked to the contraction of the cells. Blocking L or T Ca^2+^ plasma membrane channels had no effect on ET-1 stimulated contraction. While ET-1 has been shown to activate the VOC channels in smooth muscle cells from porcine coronary arteries and has also been shown to augment Ca^2+^ channel currents in the smooth muscle cell membrane of the guinea pig portal vein [4, 66]. In the thoracic aorta vascular smooth muscle cells, ET-1 produces a sustained increase in [Ca^2+^]_i_ levels from voltage regulated plasma membrane channels [4, 67]. Our results in fact point to a need for the regulation of [Ca^2+^]_i_ influx in PAH that must go well beyond the use of calcium channel blockers. Clearly, understanding the source of [Ca^2+^]_i_ should facilitate its modulation in patients with the disease.

Taken together this study provides new information for future strategies toward the modulation of the vascular hypercontraction often seen in PAH. One such strategy addresses the role of caldesmon activity, which could prove a key regulatory point in the excessive pulmonary arterial constriction. Other findings herein suggest strategies should be applied towards greater inhibition of ROCK1/2 and of Ca^2+^ influx from the cellular compartments of the HPASMC.

## Supporting information

S1 Fig Time lapse video of HPASMC treated with 100uM ET-1 over 40 min. Arrow pointing to cell responding to ET-1 over 40 min. Images were taken at time 0 of ET-1 addition and every 5 min after for 40 min. This video shows a non-PAH (Control-1) HPASMC and is representative of a typical ET-1 response.
(WMV)

S2 Fig Comparison of ET-1 induced contraction between non-PAH and PAH HPASMC. Electrical impedance was measured as described in the methods section. Triplicate wells were treated with or without (CON) 100 nM ET-1 in a non-PAH (A), hereditary PAH (B), or idiopathic PAH HPASMC (C). The line graphs represent the average normalized cell index across triplicate wells and the error bars are the standard deviation at each time point. These graphs were representative of typical ET-1 contraction responses from HPASMC Control-1 (A), PAH-1 (B), and PAH-3 (C).
(TIF)

S1 Table List of HPASMC donor information. List of gender, age and possible germline mutations of HPASMC donors.
^a^Lonza CC2581
^b^Cell Applications Inc. #1487.
(DOCX)
