# Supplementary figures and images for: Unraveling endothelin-1 induced hypercontractility of human pulmonary artery smooth muscle cells from patients with pulmonary arterial hypertension
Source: PLoS One. 2018 Apr 12;13(4):e0195780. doi: 10.1371/journal.pone.0195780 (PMC5897024; doi:10.1371/journal.pone.0195780)

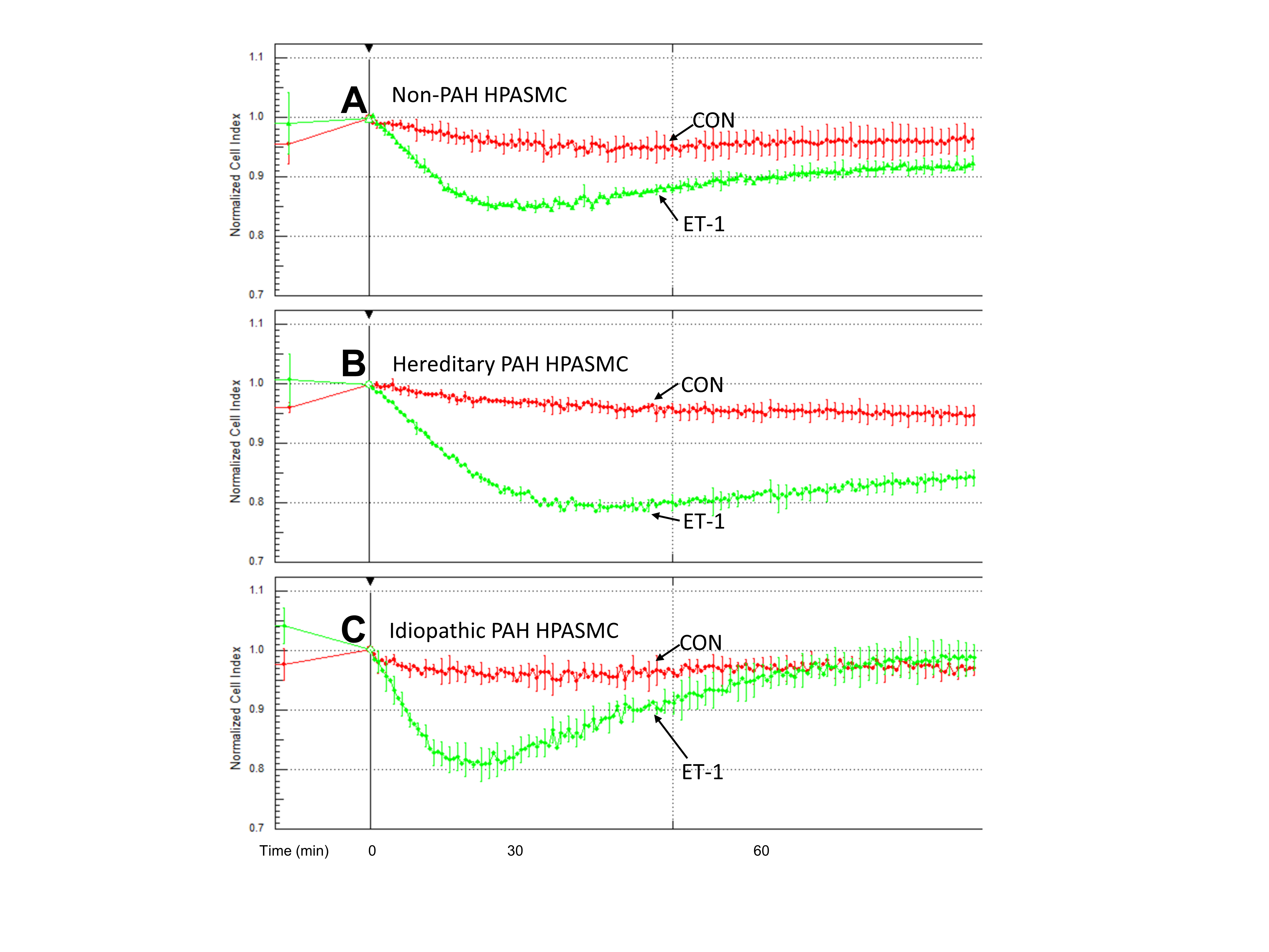

Supplement: S2 Fig — Electrical impedance was measured as described in the methods section. Triplicate wells were treated with or without (CON) 100 nM ET-1 in a non-PAH (A), hereditary PAH (B), or idiopathic PAH HPASMC (C). The line graphs represent the average normalized cell index across triplicate wells and the error bars are the standard deviation at each time point. These graphs were representative of typical ET-1 contraction responses from HPASMC Control-1 (A), PAH-1 (B), and PAH-3 (C). (TIF) [file pone.0195780.s002.tif]
