# Supplementary material for: Unraveling endothelin-1 induced hypercontractility of human pulmonary artery smooth muscle cells from patients with pulmonary arterial hypertension
Source: PLoS One. 2018 Apr 12;13(4):e0195780. doi: 10.1371/journal.pone.0195780 (PMC5897024; doi:10.1371/journal.pone.0195780)
Supplement: S1 Table — List of gender, age and possible germline mutations of HPASMC donors. aLonza CC2581 bCell Applications Inc. #1487. (DOCX) [file pone.0195780.s003.docx]

**S1 Table. List of HPASMC Donor Information**

| **Subject** | **Gender** | **Age** | **Germline Mutation** |
| --- | --- | --- | --- |
| **Donor Controls** |  |  |  |
| Control-1 | male | 36 | None |
| Control-2 | male | 39 | None |
| Control-3 | female | 48 | None |
| Control-4^a^ | male | 43 | None |
| Control-5^b^ | male | 21 | None |
| **Hereditary or Idiopathic PAH** |  |  |  |
| PAH-1 | male | 41 | BMPR2 del Exon 1-8 |
| PAH-2 | female | 50 | BMPR2 del Exon 4-5 |
| PAH-3 | male | 42 | None |
| PAH-4 | female | 34 | None |
| PAH-5 | female | 30 | None |
